# Health and Economic Burden of Running-Related Injuries in Dutch Trailrunners: A Prospective Cohort Study

**DOI:** 10.1007/s40279-016-0551-8

**Published:** 2016-05-25

**Authors:** Luiz Carlos Hespanhol Junior, Willem van Mechelen, Evert Verhagen

**Affiliations:** 1 Amsterdam Collaboration on Health and Safety in Sports, Department of Public and Occupational Health and the EMGO+ Institute for Health and Care Research, VU University Medical Center Amsterdam, Van der Boechorststraat 7, 1081 BT Amsterdam, The Netherlands; 2 School of Human Movement and Nutrition Sciences, Faculty of Health and Behavioural Sciences, University of Queensland, Brisbane, QLD Australia; 3 UCT/MRC Research Unit for Exercise Science and Sports Medicine (ESSM), Department of Human Biology, Faculty of Health Sciences, University of Cape Town, Cape Town, South Africa; 4 School of Public Health, Physiotherapy and Population Sciences, University College Dublin, Dublin, Ireland; 5 Australian Centre for Research into Injury in Sport and its Prevention, Federation University Australia, Ballarat, VIC Australia

## Abstract

**Background:**

Trailrunning is becoming very popular. However, the risk and burden of running-related injuries (RRI) in trailrunning is not well established.

**Objective:**

To investigate the prevalence, injury rate, severity, nature, and economic burden of RRIs in Dutch trailrunners.

**Methods:**

This prospective cohort study included 228 trailrunners aged 18 years or over (range 23–67), and was conducted between October 2013 and December 2014. After completing the baseline questionnaire, the Oslo Sports Trauma Research Center Questionnaire on Health Problems was administered every 2 weeks to collect data on RRIs. Participants who reported RRIs were asked about healthcare utilization (direct costs) and absenteeism from paid work (indirect costs). RRI was defined as disorders of the musculoskeletal system or concussions experienced or sustained during participation in running.

**Results:**

The mean prevalence of RRIs measured over time was 22.4 % [95 % confidence interval (CI) 20.9–24.0], and the injury rate was 10.7 RRIs per 1000 h of running (95 % CI 9.4–12.1). The prevalence was higher for overuse (17.7 %; 95 % CI 15.9–19.5) than for acute (4.1 %; 95 % CI 3.3–5.0) RRIs. Also, the injury rate was higher for overuse (8.1; 95 % CI 6.9–9.3) than for acute (2.7; 95 % CI 2.0–3.4) RRIs. The median of the severity score was 35.0 [25–75 %, interquartile range (IQR) 22.0–55.7], and the median of the duration of RRIs was 2.0 weeks (IQR 2.0–6.0) during the study. The total economic burden of RRIs was estimated at €172.22 (95 % CI 117.10–271.74) per RRI, and €1849.49 (95 % CI 1180.62–3058.91) per 1000 h of running. An RRI was estimated to have a direct cost of €60.92 (95 % CI 45.11–94.90) and an indirect cost of €111.30 (95 % CI 61.02–192.75).

**Conclusions:**

The health and economic burden of RRIs presented in this study are significant for trailrunners and for society. Therefore, efforts should be made in order to prevent RRIs in trailrunners.

**Electronic supplementary material:**

The online version of this article (doi:10.1007/s40279-016-0551-8) contains supplementary material, which is available to authorized users.

## Key Messages

**Table:** 

At any given time, one in five trailrunners report having a running-related injury (RRI).
Of the RRIs in trailrunners, 75.2 % were overuse injuries, and the prevalence of overuse RRIs was fourfold higher than acute RRIs.
The indirect cost of RRIs (related to absenteeism from paid work) was twofold higher than the direct cost (related to healthcare utilization).

## Introduction

Physical activity is a cost-effective and cost-saving intervention to improve overall health and gain healthy life-years [1–4]. There is evidence claiming that physical activity participation in outdoor environments has a larger beneficial effect on physical and mental wellbeing than participation in indoor physical activities [5]. Coincidentally, trailrunning, a mode of running consisting of running in the outdoors on unpaved and hilly/mountain terrains, is quickly gaining in popularity worldwide. The trailrunning community is composed of well trained trailrunners who participate in ultra-marathon events (>42.2 km), but also increasingly by trailrunning enthusiasts who partake in trailrunning events with shorter distances.

Running is a very popular mode of exercise among people seeking an active lifestyle [6, 7]. Next to being beneficial for health [8–10], running also carries a risk of running-related injuries (RRI) with incidence rates ranging from 7.7 [95 % confidence interval (CI) 6.9–8.7] to 17.8 (95 % CI 16.7–19.1) RRIs per 1000 h of running in recreational and novice runners, respectively [11]. However, prospective data on the risk and burden (including costs) of RRIs in trailrunning are sparse, especially in cohorts including trailrunning enthusiasts that compose the general trailrunning population.

Most RRIs have an overuse nature [12] of which the symptoms can last for several weeks [13]. Also, these injuries can negatively influence physical activity participation [14, 15]. Consequently, measuring overuse injuries next to acute injuries is important to understand the overall burden of RRIs [15]. However, measuring overuse injuries is challenging, because of their non-identifiable and gradual onset, and also due to fluctuation of symptoms over time [16]. Most studies about running have measured RRIs leading to consequences, such as time loss (i.e. running sessions not fully accomplished or completely missed due to RRIs) and/or medical attention [17]. Defining RRI based only on these consequences could underestimate the overall burden of RRIs, since minor injuries not resulting in such consequences would be neglected [18, 19]. Also, to register overuse injuries accurately, one needs a long follow-up time including regular measurement intervals in order to chart the gradual onset and fluctuations of symptoms related to overuse RRIs [16, 18]. Such data are sparse in the RRI literature, and completely missing in trailrunning.

The purpose of this study was therefore to prospectively investigate the prevalence, injury rate, severity, nature, and economic burden of acute and overuse RRIs in Dutch trailrunners. Such data may assist in the development of RRI prevention programs in this mode of running, and also may assist in decisions related to allocation of public health financial resources.

## Methods

### Participants

This study was composed of a convenience sample of the general Dutch trailrunning population. Individuals engaged in trailrunning were invited to partake in the study via flyer cards distributed during trailrunning events in The Netherlands, and also by social media channels, newsletters, and the MudSweatTrails (MST) website [20]. The flyer cards and additional recruitment sources guided the individuals to the project’s website containing further information and the option to enroll in the study. Individuals who agreed to participate through online informed consent, aged 18 years or over, reported running on unpaved surfaces on a regular basis, and who completed the baseline questionnaire were included in the study. A sample size calculation a priori was not possible because of a lack of information on the prevalence of RRIs repeatedly measured over time at the commencement of this study. The study was approved by the medical ethics committee of the VU University Medical Center Amsterdam, The Netherlands.

### Study Design

This was a prospective open cohort study conducted between October 2013 and December 2014. This cohort was composed of a dynamic sample, i.e., the participants entered into the study at different time-points and, therefore, they had different follow-up periods. However, all participants were followed for at least 6 months. After giving informed consent, a link to a secure online baseline questionnaire was sent by e-mail to the participants. This questionnaire asked about demographics, running experience, participation in other sports, current medical conditions, previous (last 12 months) RRIs, and current RRIs. Online follow-up questionnaires were completed every 2 weeks via a secure link sent by email. The aim of these follow-up questionnaires was to collect data about the participants’ running exposure (overall exposure and on unpaved surfaces specifically) and to record any health problems experienced in the preceding 2 weeks. In case of a sustained RRI, information about healthcare utilization and absenteeism from paid work related to the RRI were also registered through the same follow-up questionnaires (conditional branching questions). If no response was received within 1 week, a reminder was sent by e-mail encouraging the participant to complete the follow-up questionnaire.

### Health Problems Registration

In order to prospectively register health problems during the follow-up, the translated and adapted Dutch version of the Oslo Sports Trauma Research Center (OSTRC) Questionnaire on Health Problems was included in the follow-up questionnaires [21, 22]. The OSTRC questionnaire was proposed and validated to register and monitor sports-related health problems over time, i.e., acute injuries, overuse injuries, and illnesses [23]. The internal consistency (Cronbach’s *α*) of the OSTRC questionnaire was estimated at 0.96 and 0.91 for overall problems (including illnesses) and overuse injuries, respectively [21, 23].

The OSTRC questionnaire consisted of four key questions on: (1) the extent to which injury, illness, or other health problems have affected running participation; (2) running volume; (3) running performance; and (4) the extent to which the individual has experienced symptoms during the previous 2 weeks. If no problems were reported on these four key questions, the questionnaire was finished. If a problem was reported on any of the four key questions, the participant was asked to specify whether the problem was an illness or an injury. In the case of an illness, the questionnaire was finished. In case of an injury, participants were asked to report the anatomical location (one possible answer per RRI), injury type (one possible answer per RRI), a description of the symptoms (open question), injury onset, the number of days of time loss (defined as the number of training sessions not fully accomplished or completely missed due to injury), and whether the injury was related to running. In the case of multiple injuries within the fortnight, the participants were asked to register the injury that caused most complaints. Other injuries could be reported in an open question. Participants were instructed to report all problems, regardless of whether or not they had already reported the same problem in previous follow-up questionnaires.

### Classification of Health Problems

Health problems were classified as injuries if they were “disorders of the musculoskeletal system or concussions,” and were classified as illnesses if they “involved other body systems” [21]. One investigator who is also a physiotherapist (LCHJ) evaluated each reported injury case by case. Injuries were classified as RRI when they were reported as such by the participants, and when the physiotherapist confirmed that they were experienced or sustained during participation in running. Subsequently, RRIs were subcategorized into acute (the onset could be linked to a specific injury event) or overuse injuries (could not be linked to a clearly identifiable event) [21]. The Orchard Sports Injury Classification System version 10 (OSICS-10) [24] was used to provide a diagnostic classification for each RRI.

Substantial health problems were defined as those leading to moderate or major reductions in training volume, moderate or major reductions in running performance, or complete inability to run, as identified in the response options of the key questions 2 or 3 of the OSTRC questionnaire [21].

A recurrent RRI was defined as an RRI at the same location and of the same type of the index RRI, even if it concerned re-injuries (after full recovery) or exacerbations (not full recovery) [25].

### Economic Consequences of Running-Related Injuries (RRIs)

Participants who had reported an RRI were asked about their healthcare utilization (direct costs) and days of productivity loss related to paid work (indirect costs) due to RRIs for the duration of their reporting of symptoms. This information was collected through conditional branching questions in the follow-up questionnaires. The cost evaluation was performed from a societal perspective, considering all RRI-related costs regardless of who pays or benefits [26]. Table 1 provides the cost categories that were registered and related monetary costs used in this evaluation. All prices were standardized to the year 2009 according to the Dutch Health Insurance Board [27] and corrected for inflation until the year 2014 [28]. Costs of absenteeism from paid work were estimated based on the mean income [27] and working hours of the Dutch population according to age and gender [29].

**Table 1:** Monetary costs applied in the cost analysis

Description	Cost, €
Healthcare costs (direct costs)	
General practitioner (per visit, 10 min)	30.79
General practitioner (per telephone consultation)	15.40
Medical specialist (per visit)	79.17
Physiotherapist (per visit)	39.59
Costs of productivity loss (indirect costs)	
Absenteeism from paid work (per hour)*	31.22 (9.78–43.95)

Prices standardized to the year 2009 according to the Dutch Health Insurance Board [27] and adjusted for inflation until the year 2014 [28]

* Indirect costs for paid work were estimated based on the mean income [27] and working hours [29] of the Dutch population according to age and gender. The value for paid work is the mean price followed by the minimum and maximal values according to standardized prices by age and gender, adjusted for inflation [28]

### Data Analysis

Microsoft^®^ Excel^®^ 2011 version 14.5.8 (Microsoft Corporation, Redmond, WA, USA) and R version 3.2.3 (R Foundation for Statistical Computing, Vienna, Austria) were used to analyze the data. Descriptive analysis was performed to present baseline and follow-up data. Percentages were calculated for categorical variables. The mean and its 95 % CI were calculated for continuous data with Gaussian distribution, otherwise the median and the 25–75 % interquartile range (IQR) were calculated.

#### Prevalence and Injury Rate Calculations

Prevalence repeatedly measured over time is considered the preferable measure to describe the overall burden of injuries in sports involving overuse injuries [16]. The mean prevalence of RRIs repeatedly measured over time was calculated according to previous recommendations [16, 21, 23]. For each 2-week period, the prevalence was calculated by dividing the number of participants reporting RRIs during that period by the number of total questionnaire respondents during the same period. Thereafter, the mean prevalence and its 95 % CI were calculated by summing all prevalences measured every 2 weeks, divided by the number of 2-week time-periods. The injury rate was calculated by dividing the number of RRIs by the sum of total running exposure in hours [18, 30]. The number of RRIs was calculated based on the number of unique RRIs identified during the follow-up. Results were expressed as the number of RRIs per 1000 h of running and its 95 % CI.

#### Severity

In order to monitor the progress of the RRIs over time, a severity score ranging from 0 to 100 was calculated for each RRI based on the response options of the four key questions of the OSTRC questionnaire [21]. Average severity scores were calculated by taking the mean of the severity scores measured every 2 weeks for each RRI. The cumulative severity score (sum of the severity scores measured every 2 weeks) was calculated as an estimation of the total impact that each RRI had had over the course of the study. The average and cumulative time loss were also calculated for each RRI as the same manner as the severity score.

#### Costs

Mean direct, indirect, and total costs were estimated per RRI, per 1000 h of running, and per most commonly reported RRIs. The participants could present more than one RRI during the study, resulting in dependent observations. Therefore, the difference in costs between overuse and acute RRIs were estimated using linear mixed models with random intercept at the participant level, adjusted for the following possible confounders measured at baseline: age, gender, body mass index (BMI), running experience, practice of other sports, chronic condition, medication use, current RRIs, and previous RRIs. As the cost per 1000 h of running is a rate between cumulative measures at the population level (i.e., sum of costs divided by the sum of total running exposure in hours multiplied by 1000), adjustment for possible confounders was not possible. Cost data are nonparametric, therefore, 95 % CIs were obtained by bootstrapping the data with 2000 replications [31–33], as recommended for economic evaluations [26].

## Results

### Participants, Response Rate, and Running Exposure

A total of 228 trailrunners, 171 males (75.0 %) and 57 females (25.0 %), were included in the study. The baseline results are summarized in Table 2. Five male participants entered no data in the follow-up questionnaires, corresponding to an attrition rate of 2.2 %. As the participants entered in the study in different time-points, they had different follow-up periods. However, all participants were followed for at least 6 months. The median of the follow-up period was 34.0 weeks (IQR 28.0–36.0), and the response rate measured every 2 weeks was 77.3 % (IQR 57.6–88.1). The median and IQR for the weekly running exposure can be found in Table 3. On average, 22.8 % (95 % CI 20.1–25.6) of the trailrunners participated in trailrunning events every 2 weeks. The median of the distance of the trailrunning events was 28.0 km (IQR 17.5–39.1), ranging from 3 (minimum) to 230 km (maximum).

**Table 2:** Baseline data of the participants

	All participants *n* = 228	Male *n* = 171	Female *n* = 57
Age, years	43.4 (42.2–44.6)	43.8 (42.4–45.2)	42.4 (39.9–44.8)
Height, cm	178.9 (177.8–180.1)	182.4 (181.4–183.4)	168.4 (166.8–170.0)
Weight, kg	72.5 (71.1–74.0)	76.5 (75.2–77.9)	60.6 (58.9–62.2)
BMI, kg/m^2^	22.6 (22.3–22.8)	23.0 (22.7–23.3)	21.3 (20.9–21.8)
Total running experience, *n* (%)			
Up to 1 year	7 (3.1 %)	7 (4.1 %)	–
1–2 years	18 (7.9 %)	13 (7.6 %)	5 (8.8 %)
2–5 years	43 (18.9 %)	35 (20.5 %)	8 (14.0 %)
More than 5 years	160 (70.2 %)	116 (67.8 %)	44 (77.2 %)
Trailrunning experience, *n* (%)			
Up to 6 months	22 (9.6 %)	16 (9.4 %)	6 (10.5 %)
6–12 months	38 (16.7 %)	31 (18.1 %)	7 (12.3 %)
1–2 years	59 (25.9 %)	38 (22.2 %)	21 (36.8 %)
2–5 years	71 (31.1 %)	56 (32.7 %)	15 (26.3 %)
More than 5 years	38 (16.7 %)	30 (17.5 %)	8 (14.0 %)
Practice of other sports, *n* (%)			
Yes	152 (66.7 %)	111 (64.9 %)	41 (71.9 %)
No	76 (33.3 %)	60 (35.1 %)	16 (28.1 %)
Chronic condition, *n* (%)			
Yes	40 (17.5 %)	27 (15.8 %)	13 (22.8 %)
No	188 (82.5 %)	144 (84.2 %)	44 (77.2 %)
Current medication use, *n* (%)			
Yes	26 (11.4 %)	16 (9.4 %)	10 (17.5 %)
No	202 (88.6 %)	155 (90.6 %)	47 (82.5 %)
Current RRI, *n* (%)			
Yes	41 (18.0 %)	33 (19.3 %)	8 (14.0 %)
No	187 (82.0 %)	138 (80.7 %)	49 (86.0 %)
Previous RRI (last 12 months), *n* (%)			
Yes	96 (42.1 %)	71 (41.5 %)	25 (43.9 %)
No	132 (57.9 %)	100 (58.5 %)	32 (56.1 %)

Continuous data are given as mean and 95 % confidence interval

*BMI* body mass index, *RRI* running-related injury

**Table 3:** Running exposure during the follow-up

	All participants *n* = 223	Male *n* = 166	Female *n* = 57
Total running exposure			
Duration (h/week)	3.5 (2.0–5.0)	3.5 (2.0–5.0)	3.5 (2.0–5.3)
Frequency (times/week)	2.5 (1.5–3.5)	2.5 (1.5–3.5)	2.5 (2.0–3.5)
Distance (km/week)	33.6 (19.5–50.0)	35.0 (20.0–50.0)	32.5 (17.5–50.0)
Running exposure on unpaved surfaces			
Duration (h/week)	1.5 (0.5–3.0)	1.5 (0.5–2.8)	1.8 (0.8–3.0)
Frequency (times/week)	1.0 (0.5–2.0)	1.0 (0.5–2.0)	1.5 (0.5–2.0)
Distance (km/week)	15.0 (6.0–28.0)	15.0 (6.0–27.5)	16.0 (7.5–30.0)

Results are given as median and 25–75 % interquartile range (IQR)

### Prevalence, Injury Rate, Severity, and Nature of RRIs

The absolute number, prevalence, injury rate, and severity measures of RRIs can be found in Table 4. A total of 148 participants (66.4 %) reported 242 RRIs during the follow-up. Of the injured participants, 68 (45.9 %) reported multiple RRIs (i.e., different OSICS-10 diagnostic classifications). The percentage of injured participants who reported other RRIs within the 2-week time-period was 4.7 % (IQR 4.0–7.2).

**Table 4:** Absolute number, mean prevalence measured over time (every 2 weeks), injury rate, and severity measures of running-related injuries (RRIs)

RRIs	Total	Overuse	Acute	Time loss	Medical attention
Overall					
Number of RRIs registered	*n* = 242	*n* = 182	*n* = 60	*n* = 174	*n* = 72
Prevalence, mean (95 % CI)	22.4 % (20.9–24.0)	17.7 % (15.9–19.5)	4.1 % (3.3–5.0)	15.1 % (14.0–16.2)	5.9 % (5.1–6.7)
Injury rate, number of RRIs per 1000 h of running (95 % CI)	10.7 (9.4–12.1)	8.1 (6.9–9.3)	2.7 (2.0–3.4)	7.7 (6.6–8.9)	3.2 (2.4–3.9)
Severity measures, median (IQR)					
Average severity score	35.0 (22.0–55.7)	31.1 (20.0–55.0)	37.0 (28.0–57.2)	43.0 (28.6–63.0)	55.0 (34.5–70.2)
Cumulative severity score	55.5 (28.0–122.0)	63.0 (25.2–122.0)	50.0 (33.8–116.0)	78.0 (37.0–165.0)	132.0 (66.0–278.0)
Average time loss, days	2.0 (0.0–4.7)	2.0 (0.0–4.5)	2.8 (1.0–5.1)	3.3 (1.8–6.0)	4.0 (1.5–7.3)
Cumulative time loss, days	3.0 (0.0–10.0)	3.0 (0.0–10.0)	3.5 (1.0–8.0)	5.0 (3.0–15.5)	12.0 (3.0–28.2)
Duration, weeks	2.0 (2.0–6.0)	4.0 (2.0–6.0)	2.0 (2.0–4.0)	4.0 (2.0–6.0)	6.0 (3.5–10.0)
Substantial					
Number of RRIs registered	*n* = 131	*n* = 94	*n* = 37	*n* = 120	*n* = 58
Prevalence, mean (95 % CI)	9.9 % (9.1–10.8)	7.3 % (6.5–8.0)	2.3 % (1.4–3.1)	9.4 % (8.6–10.2)	3.7 % (3.1–4.3)
Injury rate, number of RRIs per 1000 h of running (95 % CI)	5.8 (4.8–6.8)	4.2 (3.3–5.0)	1.6 (1.1–2.2)	5.3 (4.4–6.3)	2.6 (1.9–3.3)
Severity measures, median (IQR)					
Average severity score	54.5 (39.9–68.3)	54.5 (39.7–68.8)	51.0 (41.2–67.3)	54.8 (41.1–69.2)	59.6 (44.1–76.6)
Cumulative severity score	109.0 (66.0–198)	113.0 (71.2–230.5)	80.0 (50.0–159.0)	117.5 (66.0–226.0)	168.0 (80.0–287.2)
Average time loss, days	4.0 (2.0–6.8)	4.0 (2.0–6.9)	4.0 (2.0–6.0)	4.2 (2.8–7.0)	5.0 (3.0–8.3)
Cumulative time loss, days	7.0 (4.0–20.0)	8.5 (4.0–23.8)	5.0 (3.0–16.0)	9.5 (4.0–21.5)	14.0 (4.0–31.5)
Duration, weeks	4.0 (2.0–8.0)	5.0 (2.0–9.5)	4.0 (2.0–6.0)	4.0 (2.0–8.0)	6.0 (4.0–10.0)

Substantial RRIs were defined as those leading to moderate or major reductions in training volume, moderate or major reductions in running performance, or complete inability to run

*95* *% CI* 95 % confidence interval, *IQR* 25–75 % interquartile range

The mean prevalence of RRIs measured every 2 weeks was 22.4 % (95 % CI 20.9–24.0). For males, the mean prevalence of RRIs was 23.0 % (95 % CI 21.3–24.7), and for females this was 20.7 % (95 % CI 18.2–23.2), with a mean difference of 2.3 percentage points (95 % CI −1.0 to 5.6). The mean prevalence of RRIs was higher for overuse than for acute RRIs, with a mean difference of 13.6 percentage points (95 % CI 10.3 to 16.9).

The injury rate was 10.7 RRIs per 1000 h of running (95 % CI 9.4–12.1). For males, the injury rate was 11.3 (95 % CI 9.7–12.9), and for females this was 9.1 (95 % CI 6.6–11.6), with an injury rate difference of 2.2 RRIs per 1000 h of running (95 % CI −0.7 to 5.1). The injury rate was higher for overuse than for acute RRIs, with an injury rate difference of 5.4 RRIs per 1000 h of running (95 % CI 4.1 to 6.8).

A total of 54.1 % (*n* = 131) of the RRIs were classified as substantial (i.e., leading to moderate or major reductions in training volume, moderate or major reductions in running performance, or complete inability to run). Fifty-nine RRIs (24.4 %) neither resulted in time loss nor in medical attention. Overuse RRIs lasted longer and presented a higher cumulative severity score than acute RRIs (Table 4). The most commonly reported RRIs were Achilles tendon injury (12.8 %, *n* = 31), calf muscle injury (10.7 %, *n* = 26), knee pain undiagnosed (8.7 %, *n* = 21), and ankle sprain (7.0 %, *n* = 17). A breakdown list with all RRIs reported during this study can be found in the Electronic Supplementary Material.

### Economic Burden of RRIs

In total, 332 healthcare consultations (21 general practitioner, 47 medical specialist, and 264 physiotherapy consultations) and 102 days of productivity loss related to paid work were registered. A total (direct plus indirect) cost of €41,677.13 was calculated for the 242 RRIs. The direct cost was €14,742.39 (€569.64 related to general practitioner, €3720.99 related to medical specialist and €10,451.76 related to physiotherapy consultations) and the indirect cost was €26,934.74 (related to absenteeism from paid work).

The costs per RRI, per 1000 h of running and per most commonly reported RRIs can be found in Table 5. Overuse RRIs presented higher physiotherapy costs than acute RRIs, and acute RRIs presented higher costs related to general practitioner than overuse RRIs. There were no statistically significant differences in costs per 1000 h of running between males and females. Of the four most commonly reported RRIs, calf muscle injuries presented the highest direct and indirect costs.

**Table 5:** Economic burden of running-related injuries (RRIs) in trailrunners

	Overall	Direct cost				Indirect cost
		Total	General practitioner	Medical specialist	Physiotherapy	Absenteeism from paid work
Cost per RRI, €						
All injuries, *n* = 242	172.22 (117.10 to 271.74)	60.92 (45.11 to 94.90)	2.35 (1.08 to 4.14)	15.38 (8.51 to 27.41)	43.19 (30.96 to 60.20)	111.30 (61.02 to 192.75)
Overuse, *n* = 182	174.40 (108.52 to 302.65)	69.96 (48.18 to 102.90)	1.44 (0.51 to 3.54)	17.84 (9.57 to 31.88)	50.68 (35.02 to 72.00)	104.44 (50.88 to 205.16)
Acute, *n* = 60	165.61 (78.19 to 363.54)	33.50 (18.55 to 55.33)	5.13 (2.05 to 10.52)	7.92 (1.32 to 22.43)	20.45 (9.90 to 41.57)	132.11 (44.36 to 301.15)
Difference (overuse minus acute)^†^	16.60 (−161.98 to 179.61)	31.05 (16.31 to 76.70)*	−3.85 (−10.42 to −3.66)*	3.79 (−14.19 to 23.45)	27.43 (23.68 to 50.98)*	−14.88 (−210.93 to 99.40)
Cost per 1000 h of running, €						
All participants, *n* = 223	1849.49 (1180.62 to 3058.91)	654.22 (465.82 to 942.68)	25.28 (11.61 to 44.41)	165.13 (91.35 to 291.60)	463.81 (323.26 to 686.48)	1195.27 (635.76 to 2346.03)
Male, *n* = 166	1783.44 (1013.66 to 3321.43)	548.35 (353.32 to 966.17)	19.46 (7.41 to 37.99)	142.93 (57.17 to 290.63)	385.96 (238.25 to 631.36)	1235.09 (582.51 to 2622.02)
Female, *n* = 57	2034.97 (1032.15 to 4630.62)	951.52 (621.73 to 1630.91)	41.63 (10.41 to 104.08)	227.45 (80.28 to 454.90)	682.44 (398.56 to 1137.39)	1083.45 (279.82 to 3134.15)
Difference (males minus females)	−251.52 (−2671.36 to 1380.54)	−403.17 (−1107.63 to 118.15)	−22.17 (−92.86 to 10.32)	−84.52 (−339.59 to 109.56)	−296.47 (−801.50 to 50.16)	151.64 (−1751.25 to 1485.12)
Cost per most commonly reported RRIs, €						
Achilles tendon injury, *n* = 31	67.60 (30.08 to 148.10)	46.68 (19.16 to 111.10)	1.99 (0.00 to 12.32)	10.22 (0.00 to 53.23)	34.48 (13.68 to 69.77)	20.91 (0.00 to 128.43)
Calf muscle injury, *n* = 26	135.85 (49.22 to 391.91)	56.00 (23.19 to 123.85)	1.18 (0.00 to 6.58)	21.32 (5.66 to 63.18)	33.50 (11.50 to 80.89)	79.86 (0.00 to 384.14)
Knee pain undiagnosed, *n* = 21	13.20 (2.83 to 33.33)	13.20 (3.05 to 31.67)	–	–	13.20 (2.92 to 33.73)	–
Ankle sprain, *n* = 17	82.20 (8.48 to 346.78)	20.96 (5.16 to 50.47)	–	–	20.96 (5.28 to 53.78)	61.24 (0.00 to 319.48)

All costs are presented in euros (€). Mean values are followed by the bias-corrected and accelerated 95 % confidence interval estimated by bootstrapping (2000 replications)

* Significant difference between overuse and acute RRIs

^†^ The difference in costs between overuse and acute RRIs were estimated using linear mixed models with random intercept at the participant level, adjusted for the following possible confounders measured at baseline: age, gender, body mass index (BMI), running experience, practice of other sports, chronic condition, medication use, current RRIs, and previous RRIs

## Discussion

### Trailrunners and Running Exposure

The sample of the current study was composed by Dutch trailrunners who were recruited during trailrunning events, or through trailrunning channels, like the MST website [20], regardless of age, gender, running experience, competition level, or training exposure (e.g., volume and intensity). As presented in Table 3, Dutch trailrunners usually train on paved and unpaved tracks. This could be explained by the fact that most Dutch trailrunners live in city areas, and, therefore, they do not have easy and fast access to trail tracks that usually are composed by rugged, muddy, and/or mountain terrains. However, trailrunners need to train on a regular basis to be prepared for the trailrunning events that usually have longer distances (median of 28 km in the current study). Therefore, the sample of trailrunners in the current study can be considered representative of the general Dutch trailrunning population. Furthermore, the characteristics of the Dutch trailrunners who participated in this study may also be similar to recreational trailrunners in other countries.

### Prevalence and Injury Rate of RRIs

The results of this study have shown that the mean prevalence of RRIs measured every 2 weeks is between 20.9 and 24.0 % (95 % CI) in trailrunners. In other words, one out of five trailrunners may be expected to sustain RRIs during a 2-week time-period. The prevalence estimates of this study are not comparable with other studies in the literature, since this is the first study to report the prevalence of RRIs repeatedly measured over time in trailrunners. In addition, previous studies on trailrunning have used different methods and RRI definitions [34, 35]. This hampers comparisons. For example, the incidence proportion of lower limb musculoskeletal injuries (22.2 %) found during the Al Andalus Ultimate Trail 2010 held in southern Spain [35] was similar to the prevalence repeatedly measured over time reported in the current study, although these are two different measures.

Hespanhol Junior et al. [15] have used similar methods as the one used in the current study to investigate RRIs in inexperienced runners training for an event. The study design, surveillance system, RRI definition and RRI classifications were the same in both studies, although the population and the follow-up period were different. The mean prevalence of all RRIs and the mean prevalence of overuse RRIs found in the current study were lower than the mean prevalences reported by Hespanhol Junior et al. [15]. This may be explained by differences in running experience [11] and training volume [36] between these two populations.

As explained in the methods, a priori sample size calculation was not possible because of missing information on the prevalence of RRIs repeatedly measured over time in a general trailrunning population at the commencement of this study. However, the study of Hespanhol Junior et al. [15] was recently available. Therefore, a post hoc sample size calculation based on the results reported in Hespanhol Junior et al. [15] and the results of the current study was possible. The sample size was estimated based on calculations for longitudinal studies with repeated measurements [37]. The prevalence of RRIs repeatedly measured over time in the study of Hespanhol Junior et al. [15] was 30.8 % (95 % CI 25.6–36.0), and in the current study was 22.4 % (95 % CI 20.9–24.0). Considering *α* = 0.05, *β* = 0.8, 17 repeated measurements (i.e., median of 34 weeks of follow-up with repeated measurements every 2 weeks), a correlation coefficient of the repeated measurements of 0.24 (calculated in the current study for the purpose of this sample size calculation), and a response rate of 77.3 % (reported in the current study), the sample size calculation suggested a cohort of 152 participants. Based on this calculation, the sample size of the current study was appropriate. This calculation may be useful as a reference for sample size calculations for future longitudinal studies with repeated measurements on RRIs.

Comparisons of injury rates of RRIs across studies are difficult because of differences in RRI definitions [11, 17, 19]. However, the time loss injury rate in trailrunners found in the current study [7.7 RRIs per 1000 h (95 % CI 6.6–8.9)] was similar to the injury rate in recreational runners reported by Videbaek et al. [7.7 RRIs per 1000 h (95 % CI 6.9–8.7)] [11], that was summarized based on studies with time loss RRI definitions.

According to the literature, overuse RRIs occur more frequently than acute RRIs [12, 15]. The results of the current study support this observation for trailrunning, since the prevalence of overuse RRIs was fourfold higher than acute RRIs, and the injury rate of overuse RRIs was threefold higher than acute RRIs. Most of the time, running can be described as an aerobic physical activity that requires long duration exertion with few changes in movement patterns. Therefore, overuse injuries with a gradual onset mechanism resulting from repetitive micro-trauma would be more expected in trailrunning than injuries with a sudden onset.

### Severity of RRIs

Severity measures are important to understand the extent to which sports injuries affect health [38]. A strength of this study was the continuous and valid method used to monitor the severity of sports injuries, irrespective of time loss or medical attention [21]. In fact, 24.4 % of the RRIs reported in this study neither resulted in time loss nor medical attention. Therefore, the results of this study support the hypothesis that measuring RRIs based only on time loss or medical attention definitions will lead to an underestimation of the burden of RRIs.

The longer duration of overuse RRIs can explain why the cumulative severity score was higher for overuse than for acute RRIs. More than half of the RRIs were classified as substantial, meaning that they caused a moderate or major reduction in running volume or running performance, or had caused a complete inability to participate in running. This result supports the hypothesis that RRIs may reach such severity levels that they can lead to dropping out of running participation [14, 15]. The implication is that RRIs may lower the motivation to participate in running, a great ally against the burden of physical inactivity, which is a leading risk factor for the global disease burden [39] and mortality [40]. In fact, running is effective in reducing mortality and disability [8, 9]; however, the adherence to running participation is essential to reach such health benefits [9, 10].

### Economic Burden of RRIs

To the best of our knowledge, this is the first study reporting the total, direct, and indirect costs of RRIs in trailrunners. The cost per RRI in trailrunners found in the current study was €172.22 (95 % CI 117.10–271.74), which was comparable to the cost per RRI found in runners training for an event (€173.72; 95 % CI 57.17–318.76) [15] and higher than the costs per RRI found in novice runners (€83.22; 95 % CI 50.42–116.02) [41]. These cost estimates are lower than the economic burden generally reported for sports injuries in other athletic populations [42, 43]. However, comparisons with other sports and populations should be made with caution where the study methods and follow-up periods were different.

Healthcare consultations related to RRIs were threefold higher than the number of days of productivity loss related to paid work. However, the indirect cost of RRIs was twofold higher than the direct cost. Interestingly, the indirect-direct cost ratio was higher for acute RRIs (indirect cost fourfold higher than direct cost) than for overuse RRIs (indirect cost 1.5-fold higher than direct cost), indicating that the productivity loss impact may be higher for acute RRIs. Other studies have also shown higher indirect than direct costs related to sports injuries [43–47]. These results indicate that productivity loss is the main contributor to the economic burden of sports injuries, with a significant impact on societal financial resources. As such, policymakers should always take into account the direct and especially the indirect costs of sports injuries to drive their policies.

To put our results into perspective: according to MST, 7500 people participate in trailrunning events organized by them each year. Based on the results of the current study, one trailrunner runs approximately 3.5 h per week (i.e., 182 h per year). Therefore, one could expect to have a total cost related to RRIs of more than €2.5 million yearly, only accounting for trailrunners participating in the MST events. This figure represents around 0.4 % of all annual sports injury costs in The Netherlands [47]. Although not a large proportion, if RRIs in trailrunning are prevented, maybe hundreds of thousands of euros could be saved and redirected to other public health areas. This assumption shows the financial impact that RRIs in trailrunning could have for society.

There is sound evidence showing that physical activity is a cost-effective method to improve overall health, and gain healthy life-years [1–4]. Evidence also suggests that the health benefits of running outweigh the related risks and costs [4, 8–10]. Therefore, running may be advised for people who seek to improve their health by means of engaging in strenuous physical activity. Nonetheless, RRIs are a preventable side effect of such active engagement and prevention is warranted. Effective prevention of injuries will not only reduce the individual burden in terms of injury and costs, but will also improve joyful and continuing participation in running.

### Limitations

This study was composed of a convenience sample. As presented in Table 4, most RRIs reported in the current study were overuse injuries, i.e., those that have a non-identifiable and gradual onset, and also present fluctuation of symptoms over time. Consequently, the RRIs reported in the current study represent all RRIs that could be a result of running exposure on paved, unpaved, or both surfaces (the most likely assumption). The RRIs were self-reported and then classified by a healthcare professional (LCHJ) based on the RRI description given by the participants. A confirmation of the RRI diagnoses during face-to-face consultations was not possible due to logistic reasons. Data about medicines taken and diagnostic tests due to RRIs were not collected. This could have lead to an underestimation of the direct costs of RRIs. The cost analysis was an estimation based on Dutch standardized prices for healthcare utilization [27], and the mean income [27] and working hours of the Dutch population for absenteeism from paid work [29], all adjusted for inflation [28]. Despite the fact that this methodology has been accepted and recommended [26], it is important to realize that the cost results were estimated and do not represent actual costs.

## Conclusions

On average, one out of five trailrunners reported RRIs every 2 weeks. Overuse RRIs represented 75.2 % of all RRIs registered during the follow-up. A total of 54.1 % of all RRIs were classified as substantial. The economic burden (direct plus indirect costs) of RRIs was estimated at €172.22 (95 % CI 117.10–271.74) per RRI, and €1849.49 (95 % CI 1180.62–3058.91) per 1000 h of running. Healthcare utilization (direct costs) contributed to 35.4 % of these costs and absenteeism from paid work (indirect costs) to 64.6 %.

## Electronic supplementary material

Below is the link to the electronic supplementary material.

Supplementary material 1 (DOCX 119 kb)
