# Supplementary material for: Health and Economic Burden of Running-Related Injuries in Dutch Trailrunners: A Prospective Cohort Study
Source: Sports Med. 2016 May 25;47(2):367–77. doi: 10.1007/s40279-016-0551-8 (PMC5266769; doi:10.1007/s40279-016-0551-8)
Supplement: Supplementary file 1 — Supplementary material 1 (DOCX 119 kb) [file 40279_2016_551_MOESM1_ESM.docx]

**Health and economic burden of running-related injuries in Dutch trailrunners: a prospective cohort study**

Journal: Sports Medicine

Luiz Carlos Hespanhol Junior^1^, Willem van Mechelen^1,2,3,4^, Evert Verhagen^1,3,5^

1- Amsterdam Collaboration on Health and Safety in Sports, Department of Public & Occupational Health and the EMGO+ Institute for Health and Care Research, VU University Medical Center, Van der Boechorststraat 7, 1081 BT Amsterdam, the Netherlands.

2. School of Human Movement and Nutrition Sciences, Faculty of Health and Behavioural Sciences, University of Queensland, Brisbane, Australia.

3. UCT/MRC Research Unit for Exercise Science and Sports Medicine (ESSM), Department of Human Biology, Faculty of Health Sciences, University of Cape Town, South Africa.

4. School of Public Health, Physiotherapy and Population Sciences, University College Dublin, Dublin, Ireland.

5. Australian Centre for Research into Injury in Sport and its Prevention, Federation University Australia, Ballarat, Victoria, Australia.

**Corresponding author:** Luiz Carlos Hespanhol Junior ([l.hespanhol@outlook.com](mailto:l.hespanhol@outlook.com))

**Electronic Supplementary Material**

**- Running-related injuries reported during the *HealthyTrails* study -**

Table of Contents

Electronic Supplementary Material Appendix S1. OSICS-10 classification of the running-related injuries 2

Electronic Supplementary Material Appendix S2. Classification of the running-related injuries by body region 4

Electronic Supplementary Material Appendix S3. Classification of the running-related injuries by tissue damaged 5

# Electronic Supplementary Material Appendix S1

| OSICS-10 classification of the running-related injuries | | | |
| --- | --- | --- | --- |
| **OSICS-10 code** | **OSICS-10 classification** | **n** | **Percentage** |
| ATAX | Achilles tendon injury | 31 | 12.81% |
| QMYX | Calf muscle trigger points/ spasm | 26 | 10.74% |
| KZZX | Knee pain undiagnosed | 21 | 8.68% |
| AJXX | Ankle Sprains | 17 | 7.02% |
| BMGX | Buttock muscle strain | 10 | 4.13% |
| FZZX | Foot pain undiagnosed | 10 | 4.13% |
| XMLX | Muscle strain lower limb (crossing anatomical boundaries) | 9 | 3.72% |
| TMHX | Hamstring strain | 8 | 3.31% |
| FJPX | Plantar fasciitis strain | 8 | 3.31% |
| KGIX | ITB friction syndrome | 7 | 2.89% |
| QYBX | Tenoperiostitis of lower leg | 7 | 2.89% |
| FKBX | Blisters foot | 5 | 2.07% |
| KTXX | Knee Tendon Injury | 5 | 2.07% |
| QZZX | Lower leg pain undiagnosed | 5 | 2.07% |
| GZZX | Hip/Groin Pain undiagnosed | 4 | 1.65% |
| KTPT | Patellar tendinopathy | 3 | 1.24% |
| LZZX | Lumbar pain undiagnosed | 3 | 1.24% |
| KGPX | Patellofemoral pain | 3 | 1.24% |
| TMXX | Thigh Muscle strain/ Spasm/ Trigger Points | 3 | 1.24% |
| BTXX | Buttock/ Pelvis Tendon Injury | 2 | 0.83% |
| FKXX | Foot Laceration/ Abrasion | 2 | 0.83% |
| BTHT | Hamstring origin tendinopathy | 2 | 0.83% |
| LZHX | Lumbar pain with hamstring referral | 2 | 0.83% |
| AZZX | Ankle pain undiagnosed | 2 | 0.83% |
| ATXX | Ankle Tendon Injury | 2 | 0.83% |
| FTXX | Foot Tendon Injuries | 2 | 0.83% |
| TMCH | Hamstring cramping during exercise | 2 | 0.83% |
| GMXX | Hip and Groin Muscle Strain/ Tear | 2 | 0.83% |
| FSMX | Metatarsal stress fracture | 2 | 0.83% |
| AUXX | Chronic Ankle Instability | 1 | 0.41% |
| ASCX | Stress injury calcaneus | 1 | 0.41% |
| BGXX | Buttock and Pelvis Synovitis/ Bursitis | 1 | 0.41% |
| BZZX | Buttock pain undiagnosed | 1 | 0.41% |
| QMCX | Calf cramping during exercise | 1 | 0.41% |
| CMXX | Chest Muscle or Tendon strain/ spasm/ trigger points | 1 | 0.41% |
| CHXX | Chest Wall Soft Tissue Bruising/ Haematoma | 1 | 0.41% |
| QMYD | Delayed onset muscle soreness | 1 | 0.41% |
| FTET | Extensor tendinopathy in foot | 1 | 0.41% |
| ASCC | Fat pad contusion heel | 1 | 0.41% |
| FMXX | Foot Muscle Strain/ Spasm/ trigger Points | 1 | 0.41% |
| FHXX | Foot Soft Tissue Bruising/ Haematoma | 1 | 0.41% |
| WFFX | Fracture finger(s) - excl avulsion fractures | 1 | 0.41% |
| KTHX | Hamstring tendon injury | 1 | 0.41% |
| GHXX | Hip and Groin Soft Tissue Bruising/ Haematoma | 1 | 0.41% |
| GTXX | Hip and Groin Tendon Injuries | 1 | 0.41% |
| GMFI | Iliopsoas muscle strain/ tear | 1 | 0.41% |
| KCXX | Knee Cartilage Injury (Chondral/ Osteochondral/ Meniscal) | 1 | 0.41% |
| KKXX | Knee Laceration/ Abrasion | 1 | 0.41% |
| KCMX | Knee Meniscal cartilage injury | 1 | 0.41% |
| KMXX | Knee Muscle Strain/ Spasm/ Trigger Points | 1 | 0.41% |
| KHXX | Knee Soft Tissue Bruising/ Haematoma | 1 | 0.41% |
| QMXX | Lower leg muscle Injury | 1 | 0.41% |
| FJMX | Midfoot joint/ ligament sprain | 1 | 0.41% |
| FNMX | Morton's neuroma | 1 | 0.41% |
| BNPX | Piriformis syndrome/ sciatic nerve entrapment | 1 | 0.41% |
| QSFX | Stress fracture fibula | 1 | 0.41% |
| TMCX | Thigh muscle cramping suring exercise | 1 | 0.41% |
| TZZX | Thigh pain undiagnosed | 1 | 0.41% |
| TZXX | Thigh pain/ Injury Not otherwise specified | 1 | 0.41% |
| ATTT | Tibialis posterior tendinopathy | 1 | 0.41% |
| WFXX | Wrist and Hand Fractures | 1 | 0.41% |
| – | Not identified | 5 | 2.07% |
| – | Total | 242 | 100% |
| OSICS-10: Orchard Sports Injury Classification System version 10. | | | |

# Electronic Supplementary Material Appendix S2

| Classification of the running-related injuries by body region | | |
| --- | --- | --- |
| **Body region** | **n** | **Percentage** |
| Lower leg | 49 | 20.25% |
| Knee | 44 | 18.18% |
| Foot | 36 | 14.88% |
| Achilles | 31 | 12.81% |
| Pelvis/Hip/Groin | 25 | 10.33% |
| Upper leg | 23 | 9.50% |
| Ankle | 22 | 9.09% |
| Low back | 5 | 2.07% |
| Chest | 2 | 0.83% |
| Wrist/Hand | 2 | 0.83% |
| Multiple body regions | 3 | 1.24% |
| Total | 242 | 100% |

# Electronic Supplementary Material Appendix S3

| Classification of the running-related injuries by tissue damaged | | |
| --- | --- | --- |
| **Tissue damaged** | **n** | **Percentage** |
| Muscle | 67 | 27.69% |
| Tendon | 57 | 23.55% |
| Ligament | 18 | 7.44% |
| Bone | 13 | 5.37% |
| Fascia | 9 | 3.72% |
| Skin | 8 | 3.31% |
| Cartilage | 7 | 2.89% |
| Joint (multiple tissues) | 2 | 0.83% |
| Nerve | 2 | 0.83% |
| Fat pad | 1 | 0.41% |
| Unknown | 58 | 23.97% |
| Total | 242 | 100% |
